# Supplementary material for: Genome-wide identification and characterization of the AP2/ERF gene family in loblolly pine (Pinus taeda L.)
Source: PeerJ. 2024 May 21;12:e17388. doi: 10.7717/peerj.17388 (PMC11122039; doi:10.7717/peerj.17388)
Supplement: Supplemental Information 1 [file peerj-12-17388-s001.pdf]

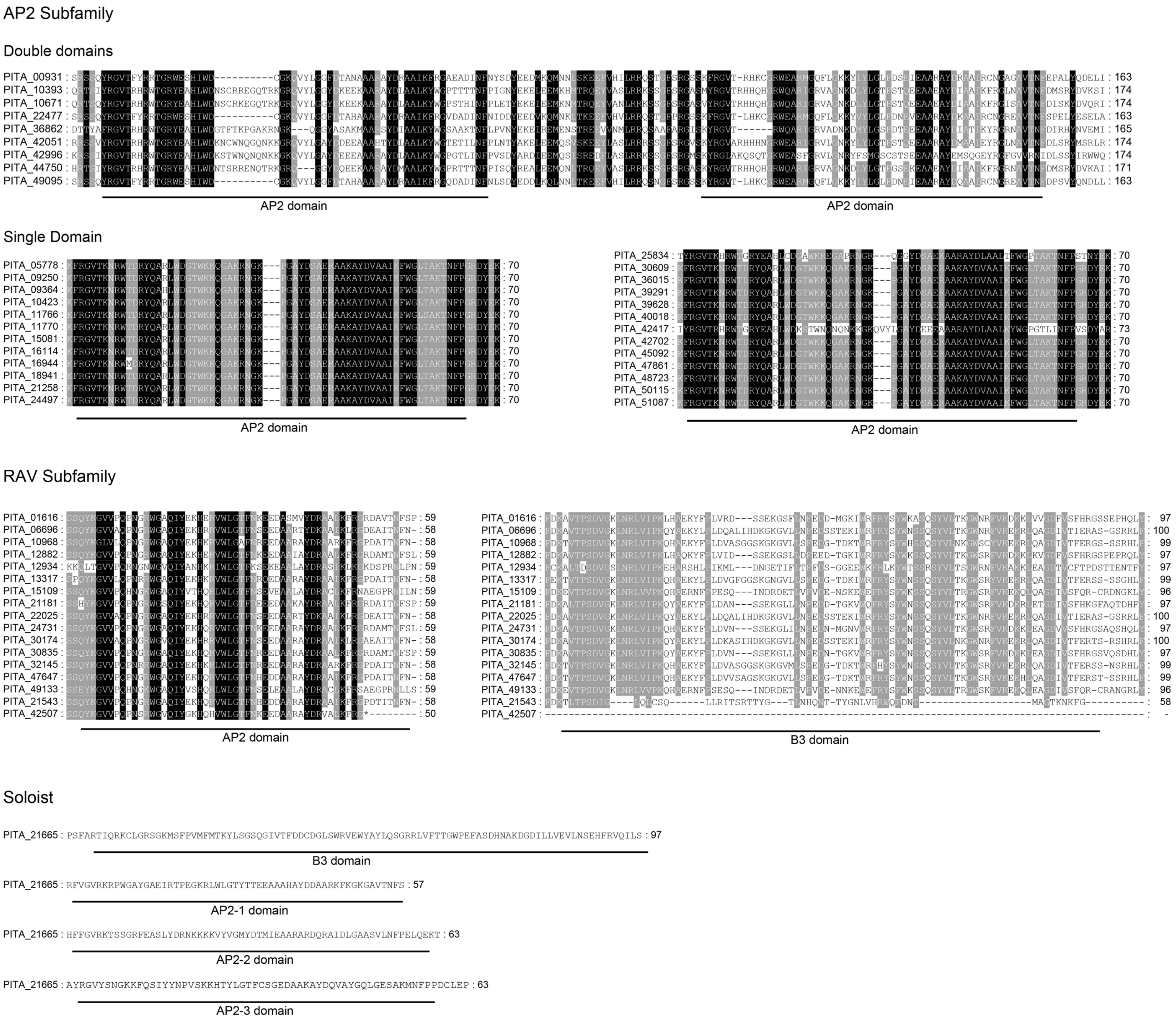

AP2 Subfamily
Double domains
AP2 domain
AP2 domain
Single Domain
AP2 domain
AP2 domain
RAV Subfamily
AP2 domain
B3 domain
Soloist
PITA_21665 : PSFARTIQRKCLGRSGKMSFPVMFMTKYLSGSQGIVTFDDCDGLSWRVEWYAYLQSGRRLVFTTGWPEFASDHNAKDGDILLVEVLNSEHFRVQILS : 97
B3 domain
PITA_21665 : RFVGVRKRPWGAYGAEIRTPEGKRLWLGTYTTEEAAAHAYDDAARKFKGKGAVTNFS : 57
AP2-1 domain
PITA_21665 : HFFGVRKTSSGRFEASLYDRNKKKKVYVGMYDTMIEAARARDQRAIDLGAASVLNFPELQEKT : 63
AP2-2 domain
PITA_21665 : AYRGVYSNGKKFQSIYYNPVSKKHTYLGTFCSGEDAAKAYDQVAYGQLGESAKMNFPPDCLEP : 63
AP2-3 domain
