# Supplementary material for: Genome-wide identification and characterization of the AP2/ERF gene family in loblolly pine (Pinus taeda L.)
Source: PeerJ. 2024 May 21;12:e17388. doi: 10.7717/peerj.17388 (PMC11122039; doi:10.7717/peerj.17388)
Supplement: Supplemental Information 2 [file peerj-12-17388-s002.pdf]

DREB Subfamily (Groups I-IV)

Double domains

```
PITA_44360 : LFKGIRMRKWGKWVSEIRVPNSKGRIWLGSYDAPEKAARAYDFAVYCLRGPEAKFNFP : 58
PITA_05232 : QFKGIRMRKWGKWVSEIRMPHSTCRIWLGSYDTPEKAARAYDFAAYCLRGSKAKLNFP : 58
PITA_44447 : QFKGTRMRKWGKWVSEIRMPHSTRRIWLGSYDTPEMAARAYDFGVYCLRGSKGKFNFP : 58
```

AP2-1 domain

```
PITA_44360 : QFKGIRMRKWGRWVSEIRIPNSKGKIWLGSYDVPEKAARAYDFAVYCLRGPEAKFNFP : 58
PITA_05232 : QFKGIRMRKWGKWVSEIRMPHSTCRIWLGSYDTPEKAARAYDFAAYCLRGSKAKLNFP : 58
PITA_44447 : QFKGIRMRKWGKWVSEIRMPHSSCRIWLGSYDTPEMAARAYDFAVYCLRGSKGKFNFP : 58
```

AP2-2 domain

Single domain

```
PITA_00259 : VYRGVRMRSWGRWVSEIREPKKKSRIWLGTFPTPELAAQAHDVATLIIKGKSTFLNFP : 58
PITA_00833 : QYKGIRMQKWGSWVCEIRMAKTREKIWLGSYKTAEQAARAYDAGVYCLKGPNAKFNLP : 58
PITA_01000 : KILGVRSAMPGKWKSEIRCG-GRQRISLGTFGRAEQAARAYDAACLALRGNSAVRNFP : 57
PITA_01074 : QFKGIRLRKWGKWVSEIRMPNCRAKIWLGSYDTPEKAARAYDFAAYCLRGSKARFNFP : 58
PITA_01484 : KYRGVKRSNRK-WTAQASNRKSKKSVHLGTFPTAEMAAVAYDAAVLVLRGESANRLFP : 57
PITA_02225 : QFKGVRMRRWGSWVSEIRAPTQKTRIWLGSYSTPEAAARAYDAALLCLKG-------- : 50
PITA_02539 : QFKGIRQRKWGSWVSEIRMPRSRKKIWLGSYTTPEQAARAYDAAV---RGRNADFNF- : 54
PITA_02556 : QFKGVRLRKWGSWVSEIRMPKSREKLWLGSYPTVEQAALAYDAAVYCLRGPTSKFNFP : 58
PITA_02581 : KILGVRSAMPGKWKSEIRCG-GRQRISLGTFGRAEQAARAYDAACLALRGNSAVRNFP : 57
PITA_02889 : KILGVRSAMPGKWKSEIRCG-GRQRISLGTFGRAEQAARAYDAACLALRGNSAMRNFP : 57
PITA_03623 : KFKGIRMRKWGKWVSEIRMPHSTRRIWLGSYDTPKMAARTYDFVMHCLKGSKAKFNFP : 58
PITA_04364 : QFKGIRMRKWGKWVSEIRMPNSRGRIWLGSYDTPEKAARAYDCAVYCLRGCKARFNFP : 58
PITA_04644 : RFRGVRRRSWGKWVAEIRMLRCRSRVWLGSYHTAEQAARAYDAASFCLRGPAAFLNFP : 58
PITA_04978 : KFKGIRLREWG----KIRIPRSREKIYLGSYKTAEQAARAFDAAMYCLRGPNAKFNFP : 54
PITA_05436 : QFKGIRLRKWGSWVAEIRMPKSREKLWLGSYKKPEQAARAYDAAVYCLRGPNAKFNLP : 58
PITA_05918 : ------MRSWGRWVSEILEPKKKSRMWLGTFPTPEMAARAHDVAALSVKGKSAFLNFP : 52
PITA_06552 : KFKGIQLREWGKWISEIRIPRSREKIYLGSYKTVEQAAQAFDAAMYCLRVPNAKFNFH : 58
PITA_06555 : LYRGVRQRHWGKWVAEIRLPRDRNRLWLGTFETAEEATLAYDRLSVLQSGVDAKLQCE : 58
PITA_06713 : VYRGLRMRSWGKWVSEIREPRKKTRIWLGTFRTPEMAARAHDVAALSVRGNSAFLNFP : 58
PITA_06934 : ------MRKWGKWVSEIRMPNSRGRIWLGSYDTPEKAARAYDCAVYCLRGCKARFNFP : 52
PITA_06985 : KILGVRSAMPGKWKSEIQCG-GRQRISLGTFGRPEQAAKAYDAACLALRGNSAVRNFP : 57
PITA_07517 : QYRGVRQRSWGKWVAEIRQPGKRTRRWLGTFATAEQAAQAYDNAAILLYGSRAHLNLQ : 58
PITA_07540 : NYRGVRQRTWGKWVSEIREPNRGARLWLGTFDTAQEAALAYDDAARALYGSDANLNLP : 58
PITA_07979 : KILGVRSAMPGKWKSEIRCG-GRQRISLGTFGRAEQAARAYDAACLALRGNSAVRNFP : 57
PITA_09315 : KILGVRSAMPGKWKSEIRCG-GRQRISLGTFGRAEQAARAYDAACLALRGNSAMRNFP : 57
PITA_09926 : KILGVRSAMPGKWKSEIRCG-GRQRISLGTFGRAEQAARAYDAACLALRGNSAVRNFP : 57
PITA_10070 : QFKGIRLREWGKWVSEIRIPRSRDKIYLGSYKTAEQAARAVAAAMYCLRGPNAKFNFP : 58
PITA_11141 : NYRGVRRRGWGKWVSEIREPRKKRRIWLGSYDTPQMAARAHDVAALCLRGKAAFLNFP : 58
PITA_13056 : KILGVRSAMPGKWKSEIRCG-GRQRISLGTFGRAEQAARAYDAACLALRGNSAVRNFP : 57
PITA_13952 : ------MRSWVKWVSEIREPRKKTRIWLGTFRTPEMAARAHDVAALSVKGNSTFLNFP : 52
PITA_14277 : PYRGVRMRKWGKWVSEVREPNKRSRIWLGSYSTPEAAARAYDTAVFYLRGPSATLNFP : 58
PITA_15245 : QFKGVRMRKWGSWGAEIRTPKTRAKIWLGSYKKAEQAARAYDAAVYCLRGPDAKFNFP : 58
PITA_15448 : QFKGIRKRKWGKWVSEIRMPNSSGRIWLGSYDTAEKAARAYDFAVYCLRGSKAKLNFP : 58
PITA_15796 : QYRGVRQRSWGKWVAEIRQPGKRTRRWLGTFATAEQAAQAYDNAAILLYGSRAHLNLQ : 58
PITA_18168 : KILGVRSSMPGKWKSEIRCG-GRQRISLGTFGRPEQAARAYDAACLALRGNSAVRNFL : 57
PITA_18762 : MYRGVRKRRWGKWVSEIREPRKKSRIWLGSFPTPEMAARAHDVAALCLKGHSAILNFP : 58
PITA_18921 : IYRGVRRRRWGKWVSEIREPRKKKRIWLGSYDTPQMAARAHDVAALCLRGKAACLNFP : 58
PITA_19406 : KILGVRSAMPGKWKSEIRCG-GRQRISLGTFGRAEQAARAYDAACLALRGNSAVRNFP : 57
PITA_21392 : VYRGLRMRSWGKWVSEIREPRKKTRIWLGTFRTPEMAA-------LSVRGNSAFLNFP : 51
PITA_22192 : GYRGVRRRSWGKWVSEIREPKKKTRIWLGSFDTPEMAARAHDVAAFHLKGKKALLNFP : 58
PITA_22354 : KFKGIRMRKWGKWVSEIRMPKSTGRIWLGSYKTAEQAARAYDAAVYCLRGPNAKFNFP : 58
PITA_22465 : PYRGVRRRNWGKWVSEIQEPKKKTRIWLGSFDTPEMAARAHDVAAFHLKGKKALLNFP : 58
PITA_22634 : KILGVRSAMPGKWKSEIRCG-GRQRISLGTFGRAEQAARAYDAACLALRGNSAVRNFP : 57
PITA_23893 : SYVGVRKRKWGSWVSEIRQPGKQSRIWLGSYSTPQEAPSAHDAAAFALRGDSAILNFP : 58
PITA_24014 : VYTGVRKRKWGKWVSEIREPKKKSRIWLGSFSTPEMAARAHDVAALCLKGPLALLNFP : 58
PITA_24062 : QFKGIRYRKWGSWVAEIRMPRSRQKLWLGSYTTAEQAARAFDAAVYCLRGPNAVFNFP : 58
PITA_24646 : QFKGIRLQEWGKWVSEI-----REKIYLGYYKTAEQAARAFDAAMYCLRGPNAKLNFP : 53
PITA_25139 : KILGVRSAMPGKWKSEIRCG-GRQRISLGTFGRAEQAARAYDAACLALRGNSAVRNFP : 57
PITA_25174 : QFKGIRLRKWGMWVSEIRMPRSRKKIWFGSYTTPEQAARAYDAAVYCLRGCNAKFNF- : 57
PITA_25177 : AYRGVRRRSWGKWVSEIKEPKKKTRTCLGSFDTPEMAARAYDVAEVYLKGKKALLNFP : 58
PITA_25473 : KYRGVKLTNRK-WRAQASYRKSKKSVHLGTFPTAEMAAVAYDAAVLVLRGESANRLFP : 57
```

AP2 domain

```
PITA_25668 : KILGVRSAMPGKWKSEIRCG-GRQRISLGTFGRAEQAARAYDAACLALRGNSAVRNFP : 57
PITA_26078 : VYRGVRMRSWGKWVSEIREPKKKSRIWLGTFPTPEMAARAHDVAALSIKGKSAFLNFP : 58
PITA_26547 : VYRGVRKRRWGKWVSEIREPKKKSRIWLGSFSTPEMAARAHDVAALCLKGHSAFLNFP : 58
PITA_26603 : -YRGVRRRRWGRWVSEIRQPGTKTRIWLGSYDKPEMAARAYDVAAVSLKGKSALPNFP : 57
PITA_26836 : PFKGIRQRKWGKWVSEVRIPNSSGRIGLGSYDTREKAARAYDFAVYCLRGSKANLNFP : 58
PITA_27078 : VYRGVRMRTWGKWVSEIREPKKKSRIWLGTFPTPEMAARAHDVAALSIKGKSAFLNFP : 58
PITA_27461 : QFKGIRMRKWGKWVSEIRMPHSTRRIWLGSYDTPERAARAYDFAAYCLRGSKAKLNFP : 58
PITA_28041 : ------MRSWGRWVSEILEPKKKSRMWLGTFPTPEMAARAHDVAALSIKGKSAFLNFP : 52
PITA_28146 : QFKGIRMRKWGKWVSEIRMPRSKERIWLGSYKTAEQAARAYDAALYCLRGPNAKFNFP : 58
PITA_28685 : QFKGIRMRKWGSWVSEIRIPKTREKIWLGSYKTAEQAARAYDAGIYCVRGPNAKFNFP : 58
PITA_30567 : KYRGVKRSNRK-WTAQASNRKSKKSVHLGTFPTAEMAAVAYDAAVLVLRGESANRLFP : 57
PITA_31683 : KILGVRSAMPGKWKSEIRCG-GRQRISLGTFGRAEQAARAYDAACLALRGNSAVRNFP : 57
PITA_31761 : QFRGIRMRKWGSWVSEIRMPKTREKIWLGSYKIPEQAARAYDAAVYFLRGPKAKFNFP : 58
PITA_32731 : KILGVRSAMPGKWKSEIRCG-GRQRISLGTFGRAEQAARAYDAACLALRGNSAVRNFP : 57
PITA_33019 : KILGVRSAMPGKWKSEIRCG-GRQRISLGTFGRPEQAAKAYDAACLALRENSAVRNFL : 57
PITA_33872 : KILGVRSAMPGKWKSEIRCG-GRQRISLGTFGRAEQAARAYDAACLALRGNSAVRNFP : 57
PITA_35223 : KILGVRSAMPGKWKSEIRCG-GRQRISLGTFGRAEQAARAYDAACLALRGNSAVRNFP : 57
PITA_36019 : KFKGIRMRKWGRWGSEIRMPKTGTKIWLGSYETAEQAARAYDAALYCLRGPNAKFNFP : 58
PITA_36346 : QFKGIRMRKWGKWVSEIRMPNSIGRIWLGSYDTPEMAARAYDFAVYCLRGSKGKFNFP : 58
PITA_38441 : LYRGVRQRHWGKWVVEIRLPRNRNRLWLGTYDTAEDAALAYDRAAYRLRGEYARLNFP : 58
PITA_38619 : QYRGVRMRSWGSWVSEIRAPHQKTRIWLGSYSTAEAAARAYDAAVLCLKGSSATLNFP : 58
PITA_38867 : KYRGVKRSNRK-WTAQASNRKSKKPVYLGTFPTAEMAAVAYDAAVLVLRGESANRLFP : 57
PITA_39167 : LYVGVRKRDWGSWVSEIRVPGKRARIWLGSYSSPEMAACAHDVAAFALRGHSAILNFP : 58
PITA_39684 : QFRGVRQRKWGKWVSEIRMPNSNGRIWLGSYDTQEKAARAYDFAVYCLRGSKVKLNFP : 58
PITA_40721 : NYRGVRQRTWGKWVAEIREPNRGSRLWLGTFSSAEEAARAYDQAARVMYGSCARLNLP : 58
PITA_41215 : QFKGIRMRKWGKWVSEIRMPRSRERIWLGSYKTAEQAARAYDAALYCLRGPNAKFNVP : 58
PITA_41382 : VYRGVRMRSWGKWVSEIREPRKKTRIWLGTFRTPEMAARAHDVAALSVRGNSAFLNFP : 58
PITA_42024 : KILGVRSAMPGKWKSEIRCG-GRQRISLGTFGRAEQAARAYDAACLALRGNSAVRNFP : 57
PITA_42265 : KYRGVKLTNRK-WRAQASYRKSKKSVHLGTFPTAEMAAVAYDAAVLVLRGESANRLFP : 57
PITA_43679 : KILGVRSAMPGKWKSEIRCG-GRQRISLGTFGRAEQAARAYDAACLALRGNSAVRNFP : 57
PITA_43769 : LLLQIQNQKWHGAVAEIRLPRNRNRLWLGTFDTAEDAALAYDRAAYRLRGEYARLNFP : 58
PITA_43933 : KYRGVKRSNRK-WTAQASNRKSKKSVHLGTFPTAEMAAVAYDAAVLVLRGESANRLFP : 57
PITA_44206 : PYRGVRRRNWGKWVSEIQEPKKKTRIWLGSFDTPEMAARAHDVAAFHLKGKKALLNFP : 58
PITA_44753 : QFKGIRLRKWGSWVAETRMPKCRQKLWLGSYTSAQQAARAYDAAVYCLRGPNAKFNFP : 58
PITA_44782 : QFKGIRLRKWGRWGSEIRVPKSRANIWLGSYETAEQAARAYDAAAYCLRGPSTEFNFP : 58
PITA_46155 : KILGVRSAMPGKWKSEIRCG-GRQRISLGTFGRAEQAARAYDAACLALRGNSAVRNFP : 57
PITA_46353 : QFKGIRMRKWGKWVSEVRIPNSSGRIWLGSYDTPEKAARAYDFAMYCLRGSTAKLNFP : 58
PITA_46383 : KYRGVKRTNRK-WRAQASYRKSKKSVHLGTFPTAEMAAVAYDAAVLVLRGESANRLFP : 57
PITA_47168 : QFKGIRLRKWGSWVAEIRMPKSREKLWLGSYKKPEQAARAYDAAVYCLRGPNAKFNLP : 58
PITA_48410 : KILGVRSAMPGKWKSEIRCG-GRQRISLGTFGRAEQAARAYDAACLALRGNSAVRNFP : 57
PITA_48797 : LYVGVRKRKWGRLVSEIRVPGKRAMIWLGSYSSPEMAACAHDVAAFALRGHSAILNFP : 58
PITA_48806 : QFKGVRMRRWGSWVSEIRAPTQKTRIWLGSYSTQEAAARAYDAALLCLKGRSASLNFP : 58
PITA_49362 : TYKGVRRRSWGKWVSEIKEPKKKRKIWLGSFDTPEMAARAYDVAEFYLKGKKALLNFP : 58
PITA_49473 : QFKGIRLRKWGKWVSEIRMPNCRAKIWLGSYESPEKAARAYDFAAYCLRGSKARFNFP : 58
PITA_50024 : VYRGLRMRSWGKWVSEIREPRKKTRIWLGTFRTPEMAARAHDVAALSVRGNSAFLNFP : 58
PITA_50199 : KYRGVKLTNRK-WRAQASYRKSKKSVHLGTFPTAEMAAVAYDAAVLVLRGESANRLFP : 57
PITA_50216 : KILGVRFAMPGKWKSEIRCG-GRQRISLGTFGRPEQAARAYDAACLALRGNSAVRNFP : 57
PITA_50272 : QFKGIRRRKWGKWVSEIRMPNSSGRIWLGSYDTPEKAARAYDFAVYCLRGSNAKLNFP : 58
PITA_50663 : KFKGIRMRKWGKWVSEIRMPKSTGRIWLGSYKTAEQAARAYDAAVYCLRGPNAKFNFP : 58
PITA_51224 : KILGVRSAMPGKWKSEIRCG-GRQRISLGTFGRAEQAARAYDAACLALRGNSAVRNFP : 57
PITA_51750 : LYRGVRQRHWGKWVAEIRLPRNRTRLWLGTFDTAEDAALAYDHEAYKLRGENARLNSP : 58
```

AP2 domain
