# Supplementary material for: Genome-wide identification and characterization of the AP2/ERF gene family in loblolly pine (Pinus taeda L.)
Source: PeerJ. 2024 May 21;12:e17388. doi: 10.7717/peerj.17388 (PMC11122039; doi:10.7717/peerj.17388)

ERF Subfamily (Groups V-X)

Double domains

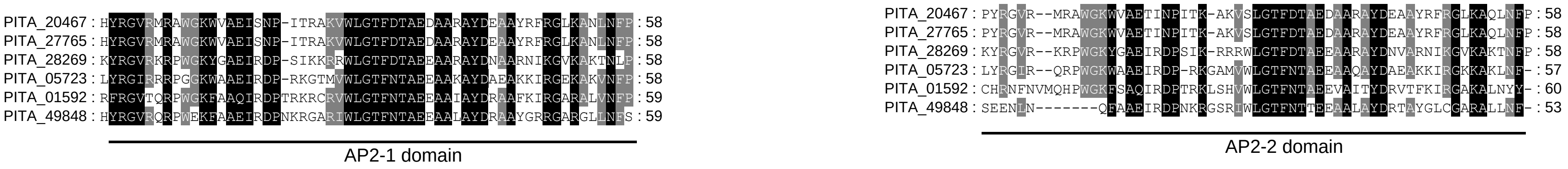

Single domain

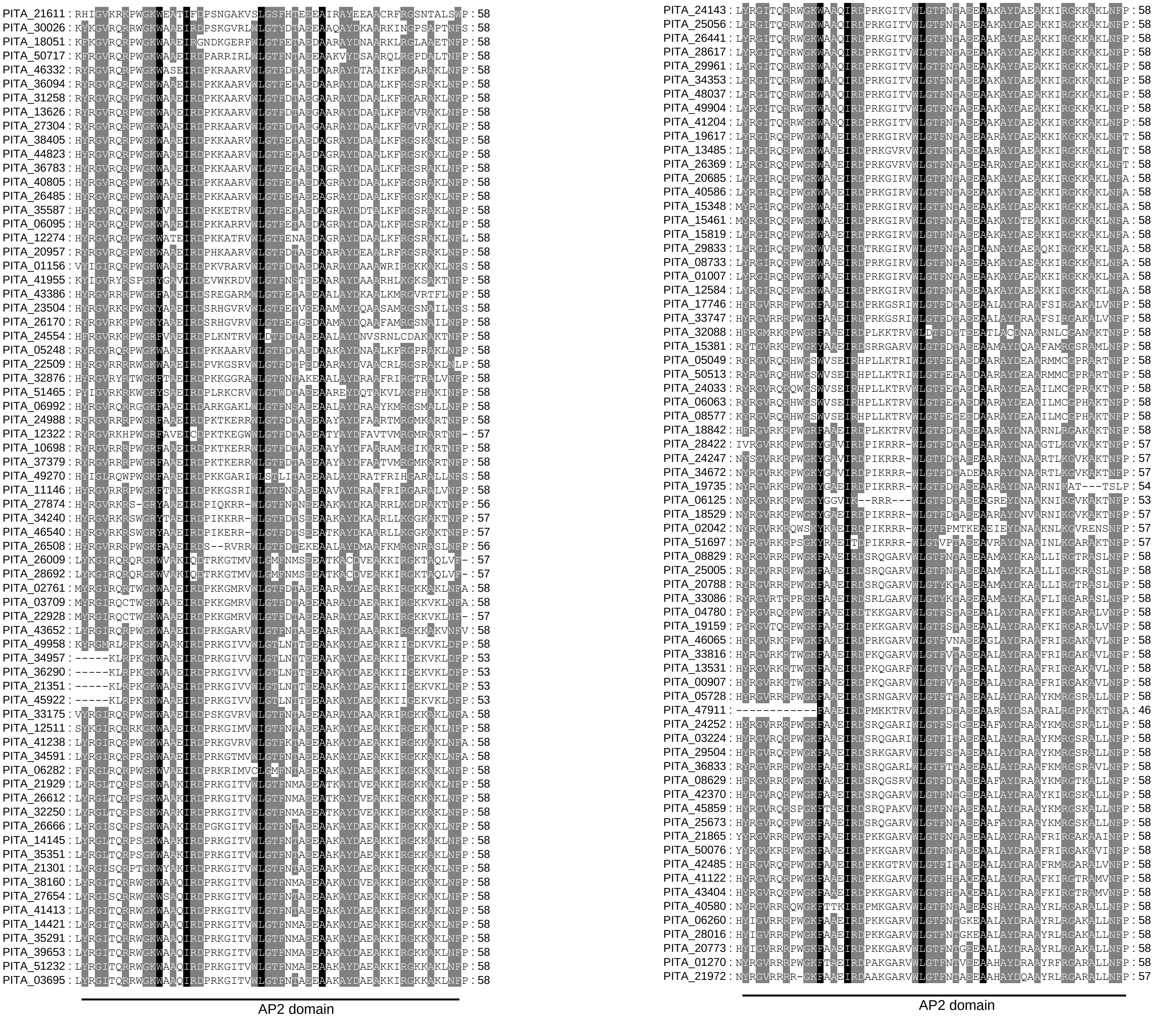

Supplement: Supplemental Information 3 [file peerj-12-17388-s003.pdf]
